# Modulating Driver Alertness via Ambient Olfactory Stimulation: A Wearable Electroencephalography Study

**DOI:** 10.3390/s24041203

**Published:** 2024-02-12

**Authors:** Mengting Jiang, Oranatt Chaichanasittikarn, Manuel Seet, Desmond Ng, Rahul Vyas, Gaurav Saini, Andrei Dragomir

**Affiliations:** 1 N.1 Institute for Health, National University of Singapore, 28 Medical Drive, #05-COR, Singapore 117456, Singapore; 2 Laboratoire des Systèmes Perceptifs, Département d’Études Cognitives, École Normale Supérieure, PSL University, CNRS, 75005 Paris, France; 3 International Operations, Procter & Gamble, 70 Biopolis Street, Singapore 138547, Singapore

**Keywords:** EEG, alertness, driving performance, road safety, olfaction, cognitive assessment

## Abstract

Poor alertness levels and related changes in cognitive efficiency are common when performing monotonous tasks such as extended driving. Recent studies have investigated driver alertness decrement and possible strategies for modulating alertness with the goal of improving reaction times to safety critical events. However, most studies rely on subjective measures in assessing alertness changes, while the use of olfactory stimuli, which are known to be strong modulators of cognitive states, has not been commensurately explored in driving alertness settings. To address this gap, in the present study we investigated the effectiveness of olfactory stimuli in modulating the alertness state of drivers and explored the utility of electroencephalography (EEG) in developing objective brain-based tools for assessing the resulting changes in cortical activity. Olfactory stimulation induced a significant differential effect on braking reaction time. The corresponding effect to the cortical activity was characterized using EEG-derived metrics and the devised machine learning framework yielded a high discriminating accuracy (92.1%). Furthermore, neural activity in the alpha frequency band was found to be significantly associated with the observed drivers’ behavioral changes. Overall, our results demonstrate the potential of olfactory stimuli to modulate the alertness state and the efficiency of EEG in objectively assessing the resulting cognitive changes.

## 1. Introduction

Road safety is critically dependent on vehicle drivers being mentally ready to react to traffic events. This entails a sufficient level of alertness, which is the heightened state of awareness to quickly detect and respond to external events, especially those that may bring about danger [1]. It has been well-documented that poor driver alertness (e.g., owing to fatigue) accounts for a sizable proportion of traffic accidents. Sleepiness can increase the risk of road accidents by a factor of 2.5 [2], accounting for approximately 6% of fatal collisions in the United States [3]. This is especially concerning for long-distance driving as the prolonged monotony increases the likelihood of vigilance decrements that could impair driving safety. Previous driving studies showed that driving performance gradually decreases with time on task, manifested behaviorally in increasing reaction times to safety critical events and accidents [4,5,6,7]. This has spurred interest to study and develop solutions to enhance driver alertness.

Alertness enhancement strategies range from techniques based on sensory stimuli, such as visual/augmented reality [8], auditory [7], haptic [9,10], or olfaction [11], to those that modulate alertness via higher-order cognitive mechanisms, such as through transcranial direct current stimulation (tDCS) [12]. While the latter technique holds potential to induce significant alertness enhancement effects, its practicality in realistic settings is limited by currently available technology and the need for an extensive closed-loop setup. Among the techniques based on sensory stimuli, olfactory stimulation has received less attention in driving scenarios, despite the extensively demonstrated effects of scents in improving performance in other types of tasks [13,14].

Olfactory stimuli are known to induce a range of physiological effects, ranging from stress relief to cognitive arousal [15]. Early work has explored how essential oils can increase heart rate and improve subjective alertness [16]. The use of environmental olfactory stimulation as a means to elevate vigilance and promote behavioral performance has been proposed [17]. Other studies demonstrated the efficiency of olfaction in enhancing spatial attention, as part of multisensory strategies for neurorehabilitation [14]. Recent work further showed the effect of scents on influencing affect in the context of technology acceptance [18], or enhancing cognitive states such as emotion [19], selective attention [20], and cognitive load [21].

Typically, modulation of cognitive states such as alertness has been assessed using subjective measures, such as questionnaires and surveys. However, such self-reported measures are often unreliable, biased, and detrimental to tasks performance, thus resulting in limited usability in real-time assessment scenarios [22]. Recent advances in wearable sensing technology have made the objective assessment of cognitive states using various behavioral and neurophysiological measures feasible and preferable, as such approaches can be seemlessly integrated within various experimental paradigms. While techniques such as eye tracking [23] and electrocardiography (ECG) [24] have been used, electroencephalography (EEG) has emerged as the most commonly used neurophysiological measure to study alertness, due to its capability to closely tap onto the neurocognitive underpinning of alertness [6,25].

Here, we first aim to investigate the modulating effect of olfactory stimuli on alertness in a monotonous driving context, which as we previously demonstrated induces significant vigilance decrement [6]. Secondly, we aim to examine the neural response underlying olfaction-modulated alertness by means of metrics estimated from the recorded EEG signals. Third, we assess the feasibility of devising an objective EEG-based classification algorithm to predict an olfaction-induced alertness state. The present research extends our previous work on tracking driving-induced vigilance and fatigue using EEG markers [4,26] and represents, to the best of our knowledge, the first effort to develop a wearable EEG-based approach to characterize olfaction-induced alertness in driving settings.

## 2. Materials and Methods

### 2.1. Participant Recruitment and Scheduling

The study protocol was approved by the Institutional Review Board of the National University of Singapore (protocol number 2022-231). Thirty-two right-handed participants between the ages of 21 to 45 were recruited, 12 female and 20 male, average age 24.5 ± 3.1 years old. The participants had no prior known sleep-deficit, neurology disorder, or smelling dysfunction, and were instructed to undergo a regular sleep cycle and avoid caffeine and alcohol consumption at least 24 h prior to the experimental time. Two experimental sessions were conducted at a one-week interval at the same time of the day for each participant (e.g., morning-morning or afternoon-afternoon sessions). Pre-screening was conducted at the beginning of the first session to determine the perceived alerting or relaxing character of two distinct fragrance stimuli. The two stimuli were subsequently used individually in the two experimental sessions for each participant in a random order: a citrus and mint-based fragrance (main components: citral and menthol) and a lavender-based fragrance (main components: linalool, linalyl acetate). These fragrances are used as components in commercial products and were prepared by a fragrance professional.

### 2.2. Experimental Procedure

**Figure 1:**
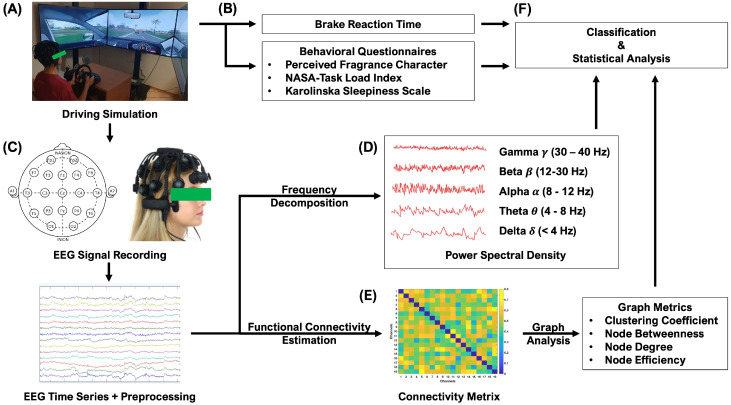
Overview of the study methodology. (**A**) Driving simulation system (**B**) Behavioral data including brake reaction time (RT) and subjective rating to verbal questionnaires (**C**) 19-channel EEG signal acquisition system and EEG signal preprocessing (**D**) EEG metrics calculation: power spectral density (PSD) in 5-frequency bands (**E**) EEG metrics calculation: graph theoretical analysis in functional brain connectivity (**F**) Classifier discriminating between fragrance’s alerting and relaxing effect and statistical analysis.

**Figure 2:**
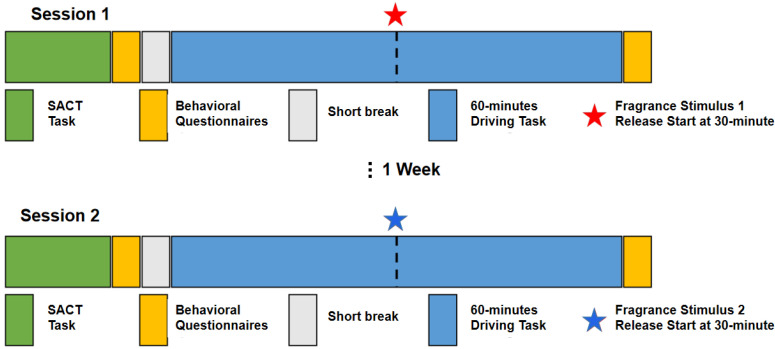
Schematic overview of the experimental procedure. Study participants underwent two experimental sessions, each consisting of the following sequence of events: SACT task to assess baseline attention state for the respective session; behavioral questionnaires (KSS); short break 10 min; 60-min driving task, during which the fragrance stimulus corresponding to that session started to be released at the minute 30 mark; behavioral questionnaires (KSS, alerting/relaxing; NASA TLX). The order of the two sessions (corresponding to the two fragrance stimuli) was randomized across subjects.

In this study, we employed a previously developed experimental procedure involving simulated driving, which we used in [6] to investigate vigilance decrement. As shown in Figure 1, in each experimental session participants were required to perform a simulated driving task while the system recorded their brake reaction time (RT) and EEG signals. Questionnaire data were collected before and immediately after the driving task (Figure 2) as follows: participants provided responses before and after the driving tasks on the Karolinska Sleepiness Scale (KSS) [27] and on the NASA Task Load Index (NASA TLX) questionnaire [28] after the driving task. Additionally, after the driving task we recorded the perceived alerting and relaxing effect of each of the fragrance stimuli using a 5-point balanced scale questionnaire (Strongly No/No/Neutral/Yes/Strongly Yes). Participants were asked to respond to two sets of three questions: “The fragrance you just smelled makes you: (1) able to concentrate; (2) feel alert; (3) feel energized” and “The fragrance you just smelled (1) makes you feel relaxed; (2) has a calming effect on you; (3) makes you feel sleepy”. The responses to the two sets of questions were summarized as the perceived alerting and relaxing effect of each fragrance stimulus, respectively, by averaging the scores of the corresponding set of questions. Furthermore, the perceived pleasantness and intensity of each stimulus (each on a 10-point scale, ranging from 0 = low to 10 = high) were recorded after the driving task.

In each session, before performing the driving task, participants were asked to perform a sustained attention to cue task (SACT) [29]. SACT results (accuracy and reaction time) were used to assess differences in the baseline attention state of each participant between the two sessions. The SACT duration was approximately 15 min, corresponding to 64 trials (as described in [29]). Statistical analysis using the Fisher’s exact test indicated a significant difference between the two sessions in both accuracy and reaction time (*p* < 0.05) for two participants and, thus, the data of these participants (one female and one male) was excluded from further analysis.

Subsequently, the driving task in each of the two sessions consisted of 60 min of monotonous driving. In each session, at minute 30 of the task, the fragrance stimulus corresponding to that session was emitted (the order of the stimuli/session were randomized to avoid odor effect). The driving simulator system consisted of the simulation software (Carnetsoft Driving Simulator; http://cs-driving-simulator.com, accessed on 11 November 2023) a driving console (Logitech G27 Racing Wheel, Lausanne, Switzerland), three 65-inch LCD screens creating an immersive driving experience, and the fragrance stimulus mounted on a ventilating fan, which resembled the ventilation system in a regular vehicle. Participants sat approximately 1.8 m in front of the screens while driving in adaptive cruise control (ACC) mode [4]. Participants were asked to maintain their lane while driving. They were advised to follow a leading car and press the brake pedal as soon as they observed the leading car’s brake light. The RT was recorded for analysis. The system maintained the distance from the lead car at approximately three car-lengths, with the ACC cruising speed kept at a maximum of 80 km/h [6]. Brake event interval ranged between 20–40 s. One brake event is a single trial, totaling approximately 115–118 trials in each session. Prior to the task, a 5-min practice drive was offered for participants to familiarize with the system. Each experimental session, including EEG preparation and questionnaire data collection, took no longer than 2 h.

### 2.3. EEG Signal Recording and Pre-Processing

EEG signals were acquired using a 19 dry-electrode CGX Quick-20 headset (Cognionics; San Diego, CA, USA) following the International 10–20 system throughout the 60-min of the driving task, with a sampling rate of 500 Hz. Each electrode impedance was kept below 2500 kΩ.

Analyses for this study were performed in MATLAB Version: 9.12.0 (R2022a). The EEGLAB toolbox and custom code were utilized for EEG signal preprocessing. The raw signals were initially resampled to 250 Hz followed by the application of a band-pass filter [1–940] Hz. The Automatic Artifact Rejection Algorithm (AAR) was then applied for noise and muscle artifacts removal. Subsequently, trial signals were extracted from the interval −10 to 0 s prior to the brake event to capture relevant cognitive brain activity, as well as to avoid motor function-dominated activity and excessive movement artifacts while participants attempted the brake. Eye blinking artifacts were then removed from the trial data using Independent Component Analysis (ICA). Preprocessed data were then grouped in non-overlapping 5-min epochs, consisting of 10 trials on average as previously described [6].

### 2.4. Feature Extraction—Brain Metrics

Brain metrics, including band power and graph theoretical metrics, were computed from preprocessed data for each trial. Specifically, the short time Fourier transform (STFT) method was used to compute power spectral density (PSD) in five frequency bands power (delta [1–4 Hz], theta [4–8 Hz], alpha [8–12 Hz], beta [12–30 Hz], and gamma [30–40 Hz]). The PSD was then estimated in each frequency band as:

(1) $$PSD=\sum_{t=t_{1}}^{t=t_{n}}\sum_{f=f_{1}}^{f=f_{2}}P_{t,f}$$

where $P_{t,f}$ represents the spectral power at time point *t* and frequency *f*, $t_{1}$ and $t_{n}$ are time boundaries of each trial data and $f_{1}$, $f_{2}$ are the frequency range of the desired frequency band.

Graph theoretical metrics were computed from the estimated functional connectivity network to quantify changes in the configuration of the functional connectivity network due to the different experimental conditions. Specifically, a phase-synchronization method, weighted Phase Lag Index (wPLI) [30], was first used to estimate the functional connectivity. Then, a sparsity threshold was applied at a ratio of 10% to 40% with increment steps of 1% to remove spurious functional network connections [31]. Subsequently, each of the four graph metrics under consideration (described in Table 1) was estimated within each frequency band by computing the area under curve (AUC) along the whole sparsity range mentioned above [32].

The four graph metrics (clustering coefficient, node degree, node betweenness, and node efficiency) represent two types of brain network properties that are crucial for achieving cognitive function [33]: (i) functional integration, and (ii) measure of functional segregation. Functional integration refers to how efficiently information is distributed across brain areas and can be indexed by the nodal degree, nodal efficiency, and nodal betweenness metrics. Functional segregation represents the extent to which brain function is segregated into local functional modules that perform specialized cognitive functions. In our case, functional segregation is quantified by the clustering coefficient metric, which is estimated as the average number of network triangles formed around each network node *i* (three nodes connected to each other) divided by the number of edges these neighboring nodes have with node *i*.

### 2.5. Statistical Analysis

To investigate the difference in the brain metrics between the two conditions in our study (i.e., the experimental sessions corresponding to the two different fragrance stimuli), Student’s *t*-test was applied for each of the estimated brain metrics. First, the metric was averaged across the trials in each epoch. The epoch before fragrance ([25–30] min) in each session was considered as the baseline epoch, and the ratios computed for each of the subsequent epochs (which corresponded to data collected under the effect of the respective fragrance stimulus) were each divided by the baseline value of the respective metric as a standardization measure (i.e., using the value of the respective metric before fragrance exposure).

For each channel and frequency band, the *t*-test was performed between the two conditions (experimental sessions corresponding to the two fragrance stimuli) on the standardized metrics values. The alternative Wilcoxon sign-rank test was adopted if the difference between two groups failed the Shapiro-Wilk test for normality. As there were multiple tests (19 channels) for each frequency band, the Benjamini–Hochberg method [34] was applied to control the false discovery rate (FDR) at the α = 0.05 level. To further investigate the neural basis of the observed behavioral differences in the two conditions due to the fragrance stimuli, we calculated the Pearson correlation coefficient between the observed differences in RT and brain metrics calculated both at each sensor.

### 2.6. Supervised Learning

The EEG power spectral density and functional connectivity metrics identified as significantly different following the statistical analysis described in Section 2.5 were then used as input for supervised learning analysis with the goal of devising an EEG-based classification algorithm to predict the olfaction-induced alertness state. To this goal, four machine learning algorithms were explored.

#### 2.6.1. *k*-Nearest Neighbors

The *k*-Nearest Neighbors (*k*NN) is a model-free classification algorithm, in which a data point newly presented to the algorithm (or test data point) is assigned the label belonging to the majority of its *k* closest labeled data points in terms of distance within the feature space. In the present study, the distance metric *d* is the Euclidean distance, and the label assignment is inversely distance-weighted. Parameter *k* was subjected to tuning using a procedure described in Section 2.6.5.

#### 2.6.2. Support Vectors Machine (SVM) with Linear Kernel

The classical SVM with linear kernel is a parametric method which identifies the linear hyperplane in the feature space that best separates classes [35]. This separating hyperplane relies on support vectors, which are representative data points in each of the classes, closest to the separating hyperplane. These support vectors define the position and the margin of the hyperplane. The *C* regularization parameter controls the tolerance of training misclassification to prevent overfitting, and in this study was subjected to tuning.

#### 2.6.3. SVM with Radial Basis Function (RBF) Kernel

When data cannot be separated by a linear hyperplane, SVMs are often implemented with the radial basis function (RBF) kernel [35]. SVM-RBF can solve non-linear separation problems by projecting the data into a higher dimensional space, in which the separation can be achieved. SVM-RBFs are also characterized by several parameters, among which are *C* (the regularization parameter) and γ (which defines the distance of the influence of a single training point from the separating hyperplane). In this study, *C* was subjected to tuning, and we set $\gamma =1/(N\times \sigma^{2})$, where *N* is the total number of features and $\sigma^{2}$ is the variance of the feature dataset.

#### 2.6.4. Extreme Gradient Boosting (XGBoost)

XGBoost is a decision-tree-based ensemble learning algorithm that relies on an optimized gradient boosting framework. Being an ensemble method, it sequentially employs several weak learning models, each enhancing the performance of the previous one, to achieve improved accuracy. XGBoost leverages additional regularizing components (gain threshold γ and L2 regularization parameter λ) that control tree building and minimize over-fitting [36] and penalize complex models. Many optimization features (e.g., approximate greedy split-finding, cache-aware access, sparsity-aware split finding) help XGBoost learn efficiently with large datasets. In this study, parameter λ was subjected to tuning, and we set γ=1 and learning rate ϵ=0.1.

#### 2.6.5. Cross-Validation and Hyperparameter Tuning

Hyperparameters were optimized for maximum classification accuracy within a cross-validation grid search framework. For *k*NN, parameter values were considered in *k*∈{1,2,3,⋯,10}. For Linear-SVM and RBF-SVM, parameter values were considered in *C*∈{0.1,1,5,10,20,50,100}. For XGBoost, parameter values were considered in λ∈{1,2,3,⋯,10}. Classifier performance was assessed via a 10-fold cross-validation procedure, which trains on a 90% dataset subsample and tests on the remaining 10% subsample in each iteration. Performance was subsequently evaluated using the classification accuracy, calculated as the average percentage of correct labels predicted by the classifier for the data points in the test set.

## 3. Results

### 3.1. Behavioral and Self-Reported Measures

#### 3.1.1. Questionnaires

**Figure 3:**
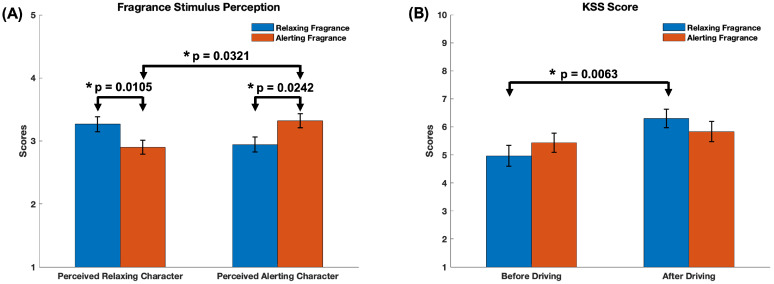
(**A**) The bar graphs illustrate the average ratings on perceived fragrance stimulus character (1 = Strongly No, 2 = No; 3 = Neutral; 4 = Yes; 5 = Strongly Yes). The ratings represent averages of the scores of the corresponding set of questions (relaxing and alerting character) for each of the two fragrance stimuli (coded here as relaxing and alerting fragrance). (**B**) the scores corresponding to the Karolinska Sleepiness Scale (KSS). 1 = Extremely alert; 10 = Extremely sleepy. Error bars represent standard error of mean. Statistical significance was determined with a significance level of *p* < 0.05 (*).

Among the two fragrance stimuli, the mint-based stimulus was predominantly perceived as more alerting and the lavender-based stimulus as more relaxing by study participants. Figure 3A displays the self-reported ratings to the two sets of questions, the relaxing character and the alerting character, summarizing the perceived alerting and relaxing effect of the two fragrance stimuli. Scores represent averages of the three responses of each set of questions.

Notably, there were statistically significant differences in the perceived relaxing character scores of the two stimuli, as the *t*-test resulted in a *t*-statistic of 2.74 with 29 degrees of freedom (t(29)=2.735,p<0.05). Similarly, there were statistically significant differences in the perceived alerting character scores with a *t*-statistic of −2.38 with 29 degrees of freedom (t(29)=−2.379,p<0.05). Thus, the fragrance stimulus perceived as more relaxing had significantly higher relaxing character scores than the stimulus perceived as alerting (M=3.267,SD=0.646 and M=2.900,SD=0.614, respectively). Similarly, the stimulus perceived as more alerting had significantly higher alerting scores than the stimulus perceived as relaxing (M=2.944,SD=0.644 and M=3.322,SD=0.603). Furthermore, the Wilcoxon signed-rank test indicated that there were statistically significant differences between the perceived alerting and relaxing character of the alerting stimulus Z=−2.143,p<0.05. There were no significant differences between the perceived intensity and pleasantness scores of the two fragrance stimuli (results shown in the Supplementary Materials Figure S1).

Figure 3B summarizes the differences in the scores of the KSS questionnaire administered before and after the driving task. The *t*-test indicated a statistically significant difference in the case of the fragrance stimulus perceived as relaxing, with a *t*-statistic of −2.94 with 29 degrees of freedom (t(29)=−2.9422,p<0.01). Thus, the mean of the KSS score after the driving task (M=6.300,SD=1.822) is significantly higher than the KSS score before the driving task (M=4.967,SD=2.076), indicating a significant increase in perceived sleepiness after the task. No difference in KSS score is observed for the fragrance stimulus perceived as alerting. Similarly, we observed no statistically significant differences in the NASA TLX scores between the two stimuli (results shown in the Supplementary Materials Figure S2).

#### 3.1.2. Behavioral Data—Reaction Times

**Figure 4:**
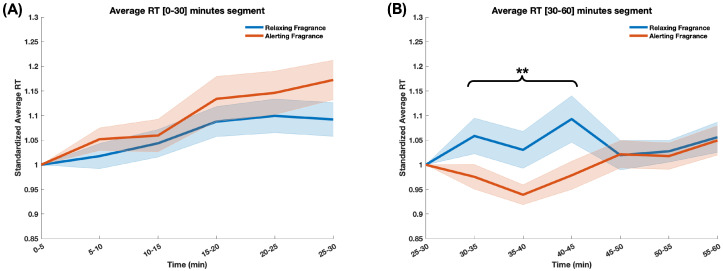
The graph illustrates changes in braking reaction time over 30-min segments: (**A**) without fragrance stimuli, during the [0–30] min segment, and (**B**) with the fragrance stimuli during the [30–60] min segment of the driving task. The two lines represent the two experimental sessions, corresponding to the two fragrance stimuli. The X-axis represents experimental time over 5 min epochs, and the Y-axis represents standardized average reaction times. Statistical significance indicates differences between the two groups during the [30–45] min segment, with a significance level of *p* < 0.01 (**). No statistically significant differences were observed in the [0–30] min segment.

As mentioned in Section 2.3 and Section 2.5, the RT data was processed in the same way as the brain metrics: first averaged across the trials in each 5-min epoch, as previously described in [6]. Subsequently, RT data in each epoch was standardized using the individual baseline value (RT in the last epoch before fragrance—[25–30 min]) to adequately account for the effect of the respective fragrance stimulus. For the [0–30] min segment, the RT data was standardized using the data of the first epoch ([0–5] min) to account for individual differences. Figure 4A shows the average RT traces during the [0–30] min segment of the driving task, and Figure 4B shows the average RT traces during the [30–60] min segment (when fragrance stimuli were delivered) for the two experimental sessions, corresponding to the exposure to the two fragrance stimuli.

The two-way repeated measure ANOVA revealed an interaction between fragrance and time (epoch) (p<0.05) over the [30–60] min of the driving task and a main effect of fragrance (p<0.01) over the [30–45] segment of the driving task. Additionally, the paired-samples *t*-test indicated that RTs in the experimental session corresponding to the relaxing fragrance were significantly higher than those of the session in which the alerting fragrance was delivered ($t\left(89\right)=-3.75,p<3\times 10^{-4}$) over the [30–45] min segment. There was no difference between the two sessions over the last 15 min of the driving task (the [45–60] segment). Similarly, there was no significant difference between the RT data in the two experimental sessions during the [0–30] min segment.

We further investigated whether age is a significant factor influencing the participants’ fragrance stimuli perception and, consequently, their RT. To this goal, we ran an analysis of covariance (ANCOVA), with age as a covariate (main factors: fragrance stimulus and experimental time/epoch; dependent variable: RT). The ANCOVA results indicated that the covariate age cannot significantly predict RT (*p* = 0.91), and there was no significant interaction effect involving age (details in the Supplementary Materials).

### 3.2. EEG Metrics—Significant Differences

To investigate the neural underpinnings of the observed behavioral differences in RT, we performed *t*-tests on each of the EEG metrics (PSD and functional connectivity), across all frequency bands within the [30–45] min segment. To this goal, the data of each trial for each subject were first averaged to obtain the values for each 5-min epoch, as described in Section 2.5. Statistical testing was then performed using the values of individual epochs across subjects. Table 2 shows the EEG metrics that were found significantly different. Of the 475 estimated metrics, a total of 108 were found to be significantly different (*p* < 0.05). Notably, the majority of the significant metrics are located in the frontal and parietal areas (76 of the 108), areas that are part of the alerting network and are known to support the ability to prepare and maintain response readiness in alertness tasks [37].

### 3.3. Classification Results

**Figure 5:**
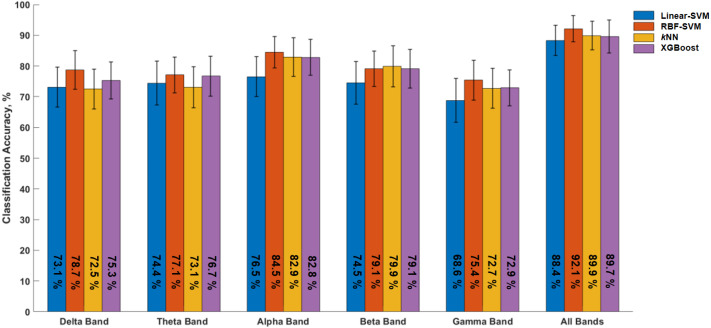
Classification accuracies obtained by the four classifiers in discriminating the altering versus relaxing state during the [30–45] min segment of the experiment using EEG metrics from each of the frequency bands and all frequency bands combined (All Bands). Delta band [1–4 Hz], theta band [4–8 Hz], alpha band [8–13 Hz], beta band [13–30 Hz], and gamma band [30–40 Hz]. Error bars represent ±1 standard error of the mean.

The significantly different EEG metrics were then used to assess their accuracy in discriminating between the two experimental conditions. Classifier models were trained on subject-level data corresponding to the metrics within individual frequency bands, as well as on the complete dataset (i.e., merging metrics from all frequency bands). The RBF-SVM (parameter C = 10) consistently yielded the highest classification accuracy scores, with the complete dataset model resulting in a classification accuracy of 92.1 ± 1.9% (Figure 5). The *k*NN (parameter *k* = 3) and XGBoost (λ = 1) classifiers achieved remarkable classification performance (89.9 ± 2.1% and 89.6 ± 2.4%, respectively), while the Linear-SVM yielded a 88.3 ± 2.6% accuracy. Among the classifiers trained on the EEG metrics within individual frequency bands, the alpha band classifiers, the highest classification accuracy was achieved by the RBF-SVM (84.5 ± 2.3%) followed by the *k*NN classifier (82.9 ± 2.8%).

The second best classification among the frequency band classifiers was obtained by the classifier trained on the beta band metrics (*k*NN, accuracy 79.9 ± 3%), while the lowest accuracy was obtained by the classifier trained on the gamma band metrics (Linear-SVM, C = 1, accuracy 68.8 ± 3.2%).

Overall, these results underline the robustness of the RBF-SVM classifier as well as the relevance of the oscillatory activity in the alpha band with respect to alertness and sustained attention-related behavior.

### 3.4. RT versus Brain Metrics—Correlational Analysis

**Figure 6:**
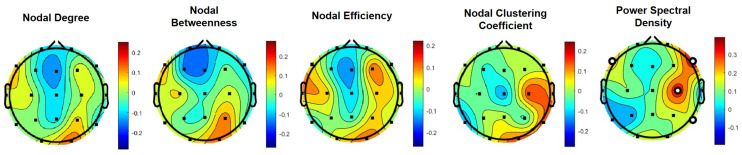
Correlational analysis on the association of driver’s behavior with EEG metrics in the alpha frequency band over the [30–45] min segment of the driving task. Topographic scalp maps indicate the correlation coefficients of braking RT and EEG metrics at all EEG channel locations. Channels with statistically significant correlation coefficients are marked with bold circles (*p* < 0.05, following FDR correction with the Benjamini–Hochberg method).

To further explore the relevance of the alpha frequency band in the observed differences in driver behavior between the two experimental conditions and to pinpoint to specific EEG metrics, correlational analysis on the association between the braking RT and individual metrics were performed. Across all EEG metrics, PSD showed the strongest correlations with braking RT over the [30–45] min segment of the driving task. Specifically, at the F8 location, the alpha band PSD was found to have a moderate correlation $(r=0.35,p<10^{-5};$ FDR corrected). The alpha band PSD at C4 location was also found to have a moderate correlation ($r=0.34,p<10^{-5};$ FDR corrected). The F7 and P8 locations were found to have weak correlations with braking RT (r=0.23 and *r* = 0.2, respectively; p<0.05; FDR corrected). Notably, as show in Table 2, the PSD values at the F7 and F8 locations were found to be significantly lower in the experimental condition corresponding to the alerting fragrance stimuli (t(89)=−3.12,p<0.01). As shown in the Figure 6 topoplots, all other alpha band EEG metrics’ correlations with braking RT did not pass statistically significant levels following false discovery rate correction using the Benjamini-Hochberg method.

## 4. Discussion

In the present study, we aimed at investigating the modulating effects of olfactory stimuli on alertness state using an experimental paradigm based on an extended assisted driving task. This 60 min monotonous driving task requires drivers to follow a lead car and brake as soon as the leading car’s brake signal is observed. Fragrance stimuli were delivered at the 30 min mark of the experiment, based on findings from our previous studies indicating continuously decreasing alertness levels as the experiment proceeds [6,26].

Alertness—also termed as vigilance or sustained attention to a task or to external stimuli over an extended time duration—is a cognitive state that is commonly studied using driving tasks. Previous works demonstrated that prolonged monotonous driving results in alertness decrement [6], manifested behaviorally in decreased reaction times to safety critical events and accidents. The decrease in alertness is caused by cognitive exertion and it is known that changes in alertness state are the result of underlying changes in the neural oscillatory activity patterns [37].

We first found that, among the two fragrance stimuli under study, the one that was subjectively perceived as more alerting by study participants induced a relative decrease in braking RT, while the stimulus perceived as more relaxing induced a relative increase in the same metric. The difference in this behavioral metric was statistically significant for ∼15 min following continuous fragrance stimuli delivery. These results suggest that the effect of the two fragrance stimuli is strongest within 15 min of exposure, after which sensory adaptation may occur, followed by subsequent cognitive and behavioral effects. This is in line with olfactory perception and adaptation studies, which indicate a timeline in the order of several minutes (depending also on odor intensity and valence) until peripheral and central adaptation occurs [38,39].

Second, we found that the observed behavioral differences are underpinned by significant changes in the neural oscillatory activity. These changes can be characterized using both univariate (estimated via power spectral density) and bivariate metrics (estimated using functional connectivity networks) computed from the EEG signals. Subsequently, using these EEG-based metrics, a panel of machine learning techniques was trained within a cross-validation scheme to assess the feasibility of objective discrimination between the neural patterns corresponding to the two experimental conditions. Among the tested classifier models, the RBF-SVM consistently yielded the best accuracy demonstrating the robustness of nonlinear models when working with metrics estimated from EEG. This is in line with findings in our previous studies on EEG-based classification of olfactory-induced changes in cognitive states [40,41]. While linear classifiers are usually desirable in brain–computer interfaces applications for their simplicity, it is often the case that with larger amounts of data, nonlinear models, and specifically nonlinear SVMs, are more likely to be more successful in deciphering complex structures in the data. This is particularly the case when working with metrics computed from EEG signals which involve nonlinear transformations of the data [42].

Third, using correlational analyses, we found statistically significant associations between the behavioral data (driving RT) and the EEG metrics, particularly in the fronto-central cortical regions, during the [30–45] min segment. The associations at the frontal locations are further supported by statistically significant differences between the PSD values in the two experimental conditions, with significantly lower alpha band PSD in the condition corresponding to the fragrance stimulus perceived as alerting, during which study participants achieved significantly lower RT values.

EEG metrics in the alpha frequency band were found to have the highest discriminative power between the two conditions, with a classification accuracy of ∼84.5%. Alpha band oscillations are known to play a major role in coalescing interactions of large-scale brain networks over different frequency ranges [43], particularly to achieve top-down control in cognitive function [44]. Moreover, alpha band activity has been linked to specific components of attention, rather than attention in general. In this context, experiments dissociating alerting versus orienting components of attention have found significant decreases in alpha band activity being linked to the alerting component [45]. Furthermore, alpha band desynchronization was explicitly linked to anticipatory attention [44,46], an aspect which is particularly relevant to our findings, since the data we examined were collected from EEG signal segments preceding the braking events. Recently, a number of neuroergonomics studies have found that frontal alpha band power decreases are more pronounced with pro-active driving, rather than re-active driving, which further underlines the relevance of this frequency band in anticipatory attention [47]. Other studies found that alpha bands at fronto-central regions are correlated with driving task performance, and may be linked to individual differences in driving performance [48], whereas higher alpha power is significantly increased in cases where the cognitive system runs in an idle state [49], or with increased mind-wandering events during the driving task [50].

### Limitations and Future Directions

The present study focused on establishing differential behavioral and brain activity effects resulting from exposure to two types of fragrance stimuli (subjectively perceived by study participants as alerting and relaxing). The behavioral effect was observed for ∼15 min following continuous exposure to the stimuli. While the effect was found to be significant, future work will need to focus on strategies to extend this effect in order to render olfactory stimulation as an effective strategy in enhancing task performance. Recent studies found that alternative delivery strategies, such as pulsed (or timed, rather than continuous exposure), as well as modulations of stimuli intensities, may limit the effects of sensory adaptation and, consequently, extend the behavioral benefits [51]. Future work may benefit from exploring different fragrance delivery techniques. Another limitation of our study stems from the use of a driving simulator with the purpose of ensuring experimental control over the behavioral aspects of the task, such as the timing of braking events, as well as general traffic environment conditions. While real-world driving conditions may differ from those in a simulator, a number of studies showed that cognitive and behavioral aspects related to driving-related hazard perception are comparable in simulated versus real driving conditions [52].

In future work we plan to additionally investigate the impact of the olfactory stimuli on motor planning and action/execution, with emphasis on the related neural mechanisms. In this direction, investigation of the role played by oscillations in the beta frequency band would be essential, as beta oscillations are known to be essential for movement preparation and execution [53]. Time-frequency analysis could reveal the differences in temporal neural dynamics, while the use of established EEG metrics based on power ratios in different frequency bands could reveal differences in underlying mental states.

## 5. Conclusions

Alertness decrement and drop in cognitive efficiency are a common occurrence when performing monotonous tasks such as driving. They result in decreased performance, increased reaction time, and may even have fatal consequences in safety-critical situations. While many studies have documented the impact of olfactory stimuli on cognitive states, relatively few studies investigated their effect on behavioral performance and the related changes in neural activity. Our present study characterized the differential effect of fragrance stimuli in modulating alertness and demonstrated that objective brain-based assessment of changes in alertness levels can be achieved using EEG-based measures. These findings hold the potential of aiding in the development of specific olfactory stimuli to mitigate alertness decrement and of facilitating the development of tools for efficient monitoring of changes in human alertness, with the goal of improving task performance and safety.

## Figures and Tables

**Table 1:** Graph metrics estimated using functional connectivity network analysis. i,j,h denote the functional network nodes (EEG sensors), *n* denotes the number of network nodes under consideration, *d* denotes the shortest path length along the network, and $t_{i}$ and $ND_{i}$ represent the number of triangles and the degree for the network node *i*, respectively.

Graph Metrics	Mathematical Formula
Nodal Degree ($ND_{i}$)	$ND_{i}$ = $\sum_{j\neq i}A_{ij}$
Nodal Betweenness ($NB_{i}$)	$NB_{i}$ = $\frac{2}{n(n-1)}\sum_{i\neq j\neq h}\frac{d_{jh(i)}}{d_{jh}}$
Nodal Efficiency ($NE_{i}$)	$NE_{i}$ = $\sum_{j\in n,j\neq i}\frac{d_{ij}^{-1}}{n-1}$
Nodal Clustering Coefficient ($CC_{i}$)	$CC_{i}$ = $\frac{2t_{i}}{ND_{i}\left(ND_{i}-1\right)}$

**Table 2:** EEG metrics found to be significantly different (*p*<0.05) between the two experimental conditions (corresponding to the two fragrance stimuli—alerting and relaxing) in the segment [30–45] min.

EEG Metric/ Channel Location	Delta Band [1–4] Hz	Theta Band [4–8] Hz	Alpha Band [4–8] Hz	Beta Band [13–30] Hz	Gamma Band [30–40] Hz
Nodal Degree	Fp1, Fp2, C3, Cz, P8, T4	Fp2, F7, F8, F4, P7, T3	F3, C4	Fp1, F4, P8, Pz, P4	P7
Nodal Betweenness	Fp1, Cz, C4, P8, T3	Fp2, F7	F3, C4, P8, Pz, O1	Fp1, P8, Pz, P4	Fp1, C4, P7
Nodal Efficiency	Fp1, Fp2, F8, C3, Cz, P8, T4	F7, F8, F4, Cz, P7, T3	F3	Fp1, F4, Pz, P4	F8, Pz, P4, O2
Nodal Clustering Coefficient	F7, C3, Pz, P3	Cz	C3, P3	Fp2, C3	F3
Power Spectral Density	F3, F8, P4, O2, T4	F3, F8, P4, O1, T4	Fp1, F7, F3, F8, Fz, F4, C3, Pz, P4, T4	Fp1, F7, F3, F8, Fz, F4, Cz, C4, P4	F7, F3, Fz, F4, C3, Cz, C4, P3

## Data Availability

Data available upon reasonable request.

## Supplementary Materials

The following supporting information can be downloaded at: https://www.mdpi.com/article/10.3390/s24041203/s1.

## Abbreviations

The following abbreviations are used in this manuscript:

AAR	Automatic Artifact Rejection
ACC	Adaptive cruise control
EEG	Electroencephalography
KSS	Karolinska Sleepiness Scale
NASA TLX	NASA Task Load Index
PSD	Power spectral density
SVM	Support Vector Machine
RBF	Radial basis function kernel
*k*NN	*k* Nearest Neighbors
RT	Reaction Time
XGBoost	Extreme Gradient Boosting
