# Supplementary material for: Modulating Driver Alertness via Ambient Olfactory Stimulation: A Wearable Electroencephalography Study
Source: Sensors (Basel). 2024 Feb 12;24(4):1203. doi: 10.3390/s24041203 (PMC10892239; doi:10.3390/s24041203)
Supplement: Supplementary file 1 [file sensors-24-01203-s001.zip › sensors-2754300-supplementary.pdf]

Supplementary Material

Perceived fragrance intensity and pleasantness were also investigated with the same method as other questionnaire components. Participants tended to perceive more pleasant and less intense when were exposed to relaxing fragrance, while participants perceived slightly stronger intensity and less pleasant the alerting fragrance. However, statistical analysis revealed that there is no significant different between relaxing and alerting fragrance on both intensity and pleasantness.

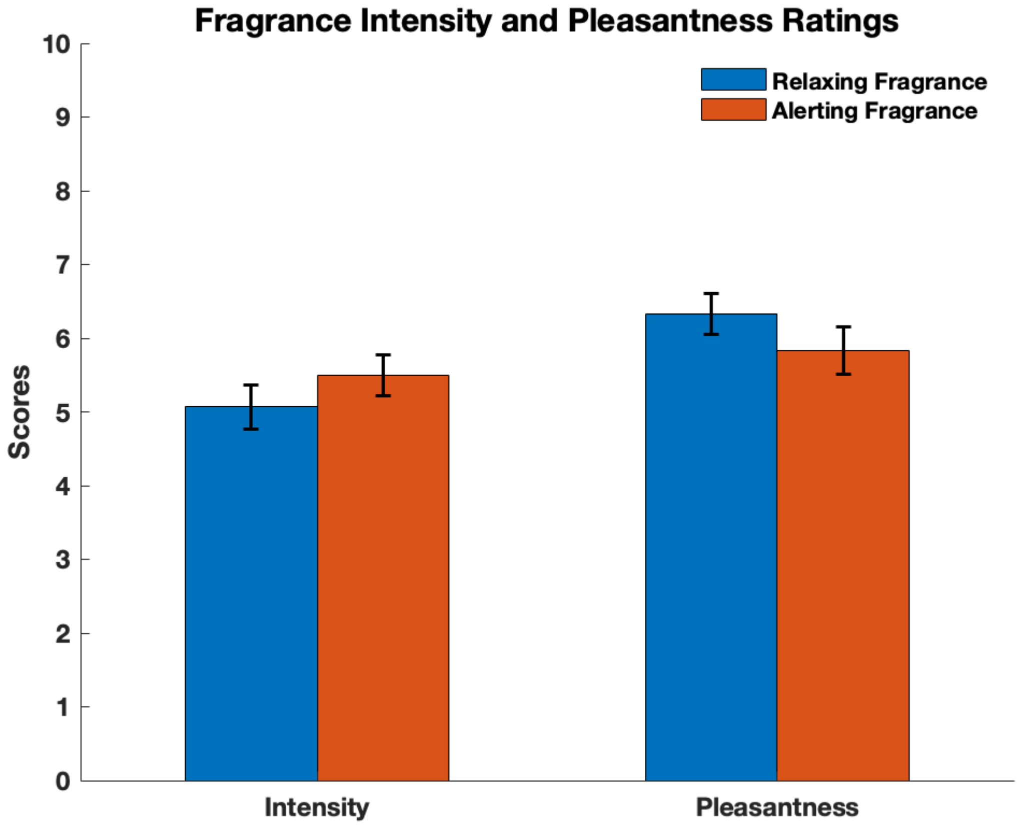

Figure S1. The bar graph illustrates the average ratings on intensity and pleasantness (0 = No smell; 2 = Can Smell; 5 = Semi Strong; 8 = Very Strong; 10 = Over whelming for intensity rating, and 0 = Poor; 5 = Good; 10 = Excellent for pleasantness rating).

NASA TLX serves as a tool for assessing the perceived workload associated with a task. Participants seem to perceive higher mental demand, effort, and frustration when they are exposed to the relaxing fragrance. However, statistical analysis revealed that there is no significant difference between relaxing and alerting fragrance on any of the individual component of NASA TLX.

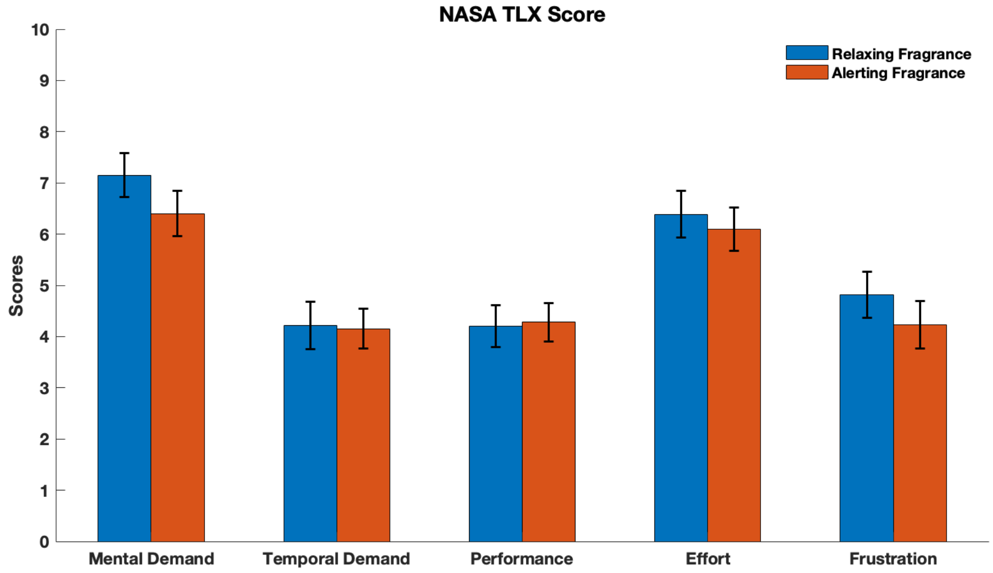

Figure S2. The bar graph illustrates the average ratings corresponding to the NASA TLX score (0 = Very Low; 10 = Very High)
